# Poly[[μ-2,2′-diethyl-1,1′-(propane-1,3-di­yl)di-1*H*-imidazole-κ^2^ *N* ^3^:*N* ^3′^](μ-5-hy­droxy­isophthalato-κ^2^ *O* ^1^:*O* ^3^)zinc]

**DOI:** 10.1107/S1600536811041456

**Published:** 2011-10-22

**Authors:** Ying-Ying Liu, Chun-Jie Wang, Yong-Sheng Yan

**Affiliations:** a Department of Chemistry and Chemical Engineering, Jiangsu University, Zhenjiang 212013, People’s Republic of China

## Abstract

In the title coordination polymer, [Zn(C_8_H_4_O_5_)(C_13_H_20_N_4_)]_*n*_, the Zn^II^ ion is coordinated by an O_2_N_2_ donor set in a distorted tetra­hedral geometry. The Zn^II^ ions are connected by 5-hy­droxy­isophthalate (hbdc) and 2,2′-diethyl-1,1′-(propane-1,3-di­yl)di-1*H*-imidazole (pbie) ligands, forming a threefold inter­penetrating diamondoid framework. In the pbie ligand, one of the ethyl­imidazole groups is disordered over two positions, with a site-occupancy ratio of 0.670 (9):0.330 (9). An inter­molecular O—H⋯O hydrogen bond is formed between the hy­droxy and carboxyl­ate groups of the hbdc ligands.

## Related literature

For background to bis­(imidazole) ligands, see: Liu *et al.* (2007 ▶, 2011 ▶).
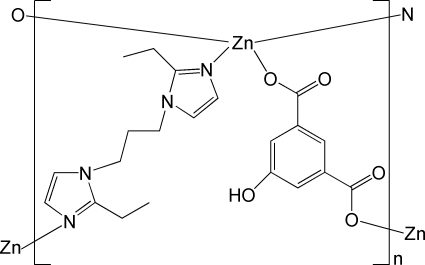


## Experimental

### 

#### Crystal data

[Zn(C_8_H_4_O_5_)(C_13_H_20_N_4_)]
*M* *_r_* = 477.83
Orthorhombic, 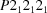

*a* = 9.476 (2) Å
*b* = 14.846 (4) Å
*c* = 15.724 (4) Å
*V* = 2212.1 (9) Å^3^
*Z* = 4
Mo *K*α radiation
μ = 1.15 mm^−1^
*T* = 293 K
0.35 × 0.28 × 0.22 mm

#### Data collection

Bruker APEX CCD diffractometer
Absorption correction: multi-scan (*SADABS*; Sheldrick, 1996 ▶) *T*_min_ = 0.68, *T*_max_ = 0.78
21558 measured reflections
5031 independent reflections
4372 reflections with *I* > 2σ(*I*)
*R*_int_ = 0.047

#### Refinement

*R*[*F* ^2^ > 2σ(*F* ^2^)] = 0.050
*wR*(*F* ^2^) = 0.136
*S* = 1.05
5031 reflections
309 parameters
15 restraints
H-atom parameters constrained
Δρ_max_ = 0.76 e Å^−3^
Δρ_min_ = −0.46 e Å^−3^
Absolute structure: Flack (1983 ▶), 2190 Friedel pairs
Flack parameter: −0.006 (19)

### 

Data collection: *SMART* (Bruker, 2007 ▶); cell refinement: *SAINT* (Bruker, 2007 ▶); data reduction: *SAINT*; program(s) used to solve structure: *SHELXS97* (Sheldrick, 2008 ▶); program(s) used to refine structure: *SHELXL97* (Sheldrick, 2008 ▶); molecular graphics: *XP* in *SHELXTL* (Sheldrick, 2008 ▶) and *DIAMOND* (Brandenburg, 1999 ▶); software used to prepare material for publication: *SHELXTL*.

## Supplementary Material

Crystal structure: contains datablock(s) global, I. DOI: 10.1107/S1600536811041456/hy2473sup1.cif

Structure factors: contains datablock(s) I. DOI: 10.1107/S1600536811041456/hy2473Isup2.hkl

Additional supplementary materials:  crystallographic information; 3D view; checkCIF report

## Figures and Tables

**Table 1:** Selected bond lengths (Å)

Zn1—O1	1.992 (3)
Zn1—O4^i^	1.950 (3)
Zn1—N1	2.021 (5)
Zn1—N1′	2.047 (13)
Zn1—N4^ii^	2.055 (4)

Symmetry codes: (i) 
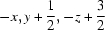
; (ii) 
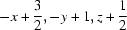
.

**Table 2:** Hydrogen-bond geometry (Å, °)

*D*—H⋯*A*	*D*—H	H⋯*A*	*D*⋯*A*	*D*—H⋯*A*
O5—H5⋯O1^iii^	0.82	1.97	2.741 (5)	156

Symmetry code: (iii) 
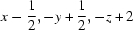
.

**Fig. 1.**
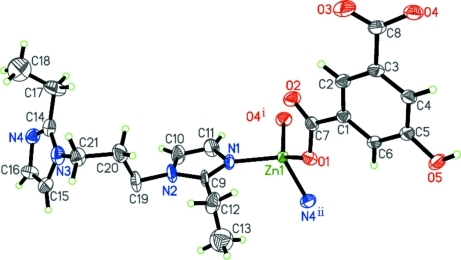
The asymmetric unit of the title compound. Displacement ellipsoids are drawn at the 30% probability level. [Symmetry codes: (i) -x, 1/2 + y, 3/2 - z; (ii) 3/2 - x, 1 - y, 1/2 + z.]

**Fig. 2.**
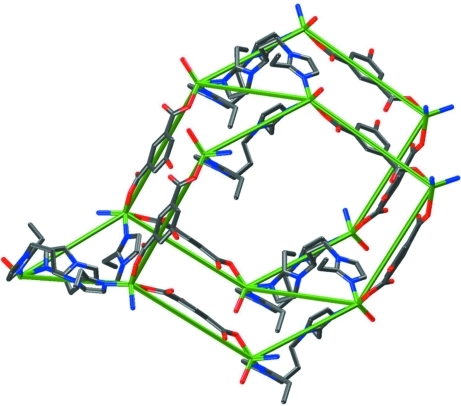
View of a single diamondiod motif.

**Fig. 3.**
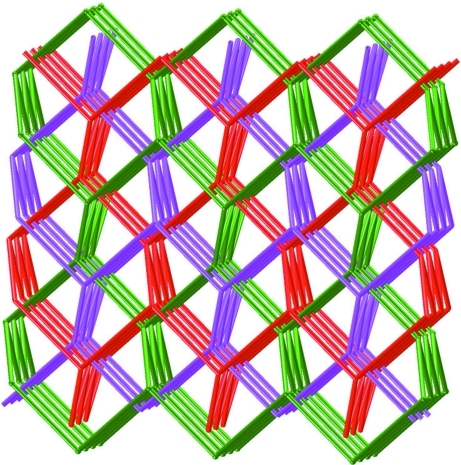
View of the threefold interpenetrating net.

## supplementary crystallographic information

### Comment

As part of an investigation of the applications of transition metal complexes, there is a need to prepare further examples of these compounds. In this paper, the structure of the title compound is described.

As shown in Fig. 1, the Zn^II^ ion is four-coordinated by two O atoms from two bridging 5-hydroxyisophthalate (hbdc) ligands and two N atoms from two bridging 1,1'-(1,3-propanediyl)bis(imidazole-2-ethyl) (pbie) ligands (Liu *et al.*, 2007, 2011). The carboxylate groups of the hbdc ligand act in a monodentate mode and the hydroxyl group is not involved in coordination. The pbie ligand coordinates to two Zn^II^ ions through its two aromatic N atoms. As illustrated in Fig. 2, the Zn^II^ ions are connected by the hbdc and pbie ligands into a diamondiod framework. To minimize the big void cavity in the diamondiod cage, a threefold interpenetrating net is generated (Fig. 3).

### Experimental

The pbie ligand was synthesized according to literature (Liu *et al.*, 2007) but 2-phenylimidazole and 1,4-dichlorobutane were replaced by 2-ethylimidazole and 1,3-dichloropropane. A mixture of ZnCO_3_ (0.050 g, 0.40 mmol), 5-hydroxyisophthalic acid (0.043 g, 0.40 mmol), pbie (0.093 g, 0.40 mmol) and water (8 ml) was sealed in a Teflon-lined reactor (15 ml) and heated at 150 °C for 3 d. After the mixture was cooled to room temperature at 10°C h^-1^, colorless crystals of the title compound were obtained (yield: 38%).

### Refinement

Disordered ethylimidazole group of the pbie ligand was refined over two sites, with a site occupancy ratio of 0.670 (9):0.330 (9). H atoms bound to C atoms were positioned geometrically and refined using a riding model, with C—H = 0.93 (aromatic), 0.97 (CH_2_) and 0.96 (CH_3_) Å and with *U*_iso_(H) = 1.2(1.5 for methyl)*U*_eq_(C). Hydroxyl H atom was refined using a riding model, with O—H = 0.82 Å and *U*_iso_(H) = 1.5*U*_eq_(O).

### Crystal data

**Table:**

[Zn(C_8_H_4_O_5_)(C_13_H_20_N_4_)]	*F*(000) = 992
*M**_r_* = 477.83	*D*_x_ = 1.435 Mg m^−^^3^
Orthorhombic, *P*2_1_2_1_2_1_	Mo *K*α radiation, λ = 0.71073 Å
Hall symbol: P 2ac 2ab	Cell parameters from 4372 reflections
*a* = 9.476 (2) Å	θ = 3.0–27.5°
*b* = 14.846 (4) Å	µ = 1.15 mm^−^^1^
*c* = 15.724 (4) Å	*T* = 293 K
*V* = 2212.1 (9) Å^3^	Block, colorless
*Z* = 4	0.35 × 0.28 × 0.22 mm

### Data collection

**Table:**

Bruker APEX CCD diffractometer	5031 independent reflections
Radiation source: fine-focus sealed tube	4372 reflections with *I* > 2σ(*I*)
graphite	*R*_int_ = 0.047
φ and ω scans	θ_max_ = 27.5°, θ_min_ = 3.0°
Absorption correction: multi-scan (*SADABS*; Sheldrick, 1996)	*h* = −12→12
*T*_min_ = 0.68, *T*_max_ = 0.78	*k* = −19→19
21558 measured reflections	*l* = −20→20

### Refinement

**Table:**

Refinement on *F*^2^	Secondary atom site location: difference Fourier map
Least-squares matrix: full	Hydrogen site location: inferred from neighbouring sites
*R*[*F*^2^ > 2σ(*F*^2^)] = 0.050	H-atom parameters constrained
*wR*(*F*^2^) = 0.136	*w* = 1/[σ^2^(*F*_o_^2^) + (0.0827*P*)^2^ + 0.6158*P*] where *P* = (*F*_o_^2^ + 2*F*_c_^2^)/3
*S* = 1.05	(Δ/σ)_max_ = 0.001
5031 reflections	Δρ_max_ = 0.76 e Å^−^^3^
309 parameters	Δρ_min_ = −0.46 e Å^−^^3^
15 restraints	Absolute structure: Flack (1983), 2190 Friedel pairs
Primary atom site location: structure-invariant direct methods	Flack parameter: −0.006 (19)

### Fractional atomic coordinates and isotropic or equivalent isotropic displacement parameters (Å^2^)

**Table:**

	*x*	*y*	*z*	*U*_iso_*/*U*_eq_	Occ. (<1)
Zn1	0.35630 (4)	0.49008 (3)	0.79285 (2)	0.03541 (14)	
C1	0.1051 (4)	0.2692 (3)	0.8438 (2)	0.0387 (9)	
C2	0.0416 (4)	0.2035 (3)	0.7938 (3)	0.0440 (8)	
H2	0.0685	0.1960	0.7373	0.053*	
C3	−0.0616 (5)	0.1496 (3)	0.8286 (3)	0.0433 (9)	
C4	−0.0976 (5)	0.1572 (3)	0.9142 (3)	0.0461 (10)	
H4	−0.1669	0.1203	0.9374	0.055*	
C5	−0.0298 (5)	0.2199 (3)	0.9643 (3)	0.0483 (10)	
C6	0.0707 (4)	0.2769 (3)	0.9292 (3)	0.0433 (9)	
H6	0.1147	0.3202	0.9627	0.052*	
C7	0.2047 (4)	0.3346 (3)	0.8022 (3)	0.0431 (9)	
C8	−0.1388 (5)	0.0841 (3)	0.7711 (3)	0.0500 (10)	
C9	0.6553 (7)	0.4354 (5)	0.7377 (5)	0.0501 (18)	0.670 (9)
C10	0.6928 (9)	0.5229 (7)	0.6298 (6)	0.074 (3)	0.670 (9)
H10	0.7355	0.5526	0.5845	0.089*	0.670 (9)
C11	0.5662 (9)	0.5394 (7)	0.6600 (6)	0.073 (3)	0.670 (9)
H11	0.5036	0.5821	0.6388	0.088*	0.670 (9)
C12	0.6801 (12)	0.3705 (7)	0.8109 (6)	0.076 (3)	0.670 (9)
H12A	0.5913	0.3418	0.8254	0.091*	0.670 (9)
H12B	0.7447	0.3238	0.7923	0.091*	0.670 (9)
C13	0.7361 (17)	0.4124 (11)	0.8850 (9)	0.125 (5)	0.670 (9)
H13A	0.7485	0.3680	0.9288	0.188*	0.670 (9)
H13B	0.6720	0.4580	0.9044	0.188*	0.670 (9)
H13C	0.8255	0.4393	0.8717	0.188*	0.670 (9)
N1	0.5406 (5)	0.4827 (5)	0.7285 (4)	0.0485 (15)	0.670 (9)
N2	0.7509 (6)	0.4549 (6)	0.6763 (5)	0.0596 (19)	0.670 (9)
C9'	0.6253 (19)	0.4478 (11)	0.6920 (11)	0.055 (4)*	0.330 (9)
C10'	0.759 (2)	0.3791 (11)	0.7897 (13)	0.065 (4)*	0.330 (9)
H10'	0.8319	0.3473	0.8158	0.078*	0.330 (9)
C11'	0.632 (2)	0.4049 (13)	0.8242 (13)	0.065 (5)*	0.330 (9)
H11'	0.6055	0.3970	0.8806	0.078*	0.330 (9)
C12'	0.582 (2)	0.4836 (13)	0.6092 (12)	0.072 (5)*	0.330 (9)
H12C	0.4805	0.4924	0.6089	0.086*	0.330 (9)
H12D	0.6050	0.4403	0.5652	0.086*	0.330 (9)
C13'	0.654 (5)	0.572 (3)	0.590 (3)	0.190 (18)*	0.330 (9)
H13D	0.6254	0.5924	0.5350	0.285*	0.330 (9)
H13E	0.7542	0.5631	0.5912	0.285*	0.330 (9)
H13F	0.6276	0.6153	0.6323	0.285*	0.330 (9)
N1'	0.5536 (13)	0.4425 (9)	0.7646 (9)	0.048 (3)*	0.330 (9)
N2'	0.7539 (16)	0.4120 (11)	0.7057 (11)	0.064 (4)*	0.330 (9)
C14	1.0407 (5)	0.3771 (3)	0.4292 (3)	0.0562 (11)	
C15	1.1817 (6)	0.3995 (4)	0.5348 (3)	0.0669 (13)	
H15	1.2243	0.3984	0.5880	0.080*	
C16	1.2190 (6)	0.4502 (4)	0.4687 (3)	0.0604 (12)	
H16	1.2954	0.4895	0.4674	0.073*	
C17	0.9295 (7)	0.3325 (5)	0.3727 (5)	0.0909 (17)	
H17A	0.8679	0.2959	0.4079	0.109*	
H17B	0.8724	0.3788	0.3461	0.109*	
C18	0.9932 (11)	0.2750 (7)	0.3056 (6)	0.131 (3)	
H18A	0.9196	0.2485	0.2719	0.196*	
H18B	1.0482	0.2282	0.3316	0.196*	
H18C	1.0528	0.3111	0.2699	0.196*	
C19	0.8787 (5)	0.4029 (5)	0.6543 (4)	0.0755 (17)	
H19A	0.9249	0.3857	0.7068	0.091*	
H19B	0.9426	0.4426	0.6240	0.091*	
C20	0.8589 (6)	0.3184 (5)	0.6009 (4)	0.0743 (15)	
H20A	0.8199	0.2713	0.6367	0.089*	
H20B	0.7908	0.3309	0.5564	0.089*	
C21	0.9938 (6)	0.2838 (4)	0.5604 (3)	0.0610 (13)	
H21A	1.0570	0.2639	0.6051	0.073*	
H21B	0.9711	0.2318	0.5256	0.073*	
N3	1.0663 (5)	0.3485 (3)	0.5086 (3)	0.0614 (10)	
N4	1.1266 (5)	0.4352 (3)	0.4023 (2)	0.0554 (9)	
O1	0.2832 (3)	0.3822 (2)	0.85403 (19)	0.0452 (7)	
O2	0.2081 (4)	0.3430 (3)	0.7259 (2)	0.0658 (10)	
O3	−0.0846 (5)	0.0604 (3)	0.7040 (3)	0.0904 (13)	
O4	−0.2584 (3)	0.0565 (2)	0.7965 (2)	0.0586 (8)	
O5	−0.0558 (4)	0.2318 (3)	1.0491 (2)	0.0723 (11)	
H5	−0.1164	0.1961	1.0647	0.109*	

### Atomic displacement parameters (Å^2^)

**Table:**

	*U*^11^	*U*^22^	*U*^33^	*U*^12^	*U*^13^	*U*^23^
Zn1	0.0326 (2)	0.0447 (2)	0.0290 (2)	0.00247 (17)	0.00191 (16)	0.00315 (17)
C1	0.040 (2)	0.043 (2)	0.0338 (18)	−0.0005 (16)	−0.0058 (15)	0.0043 (15)
C2	0.049 (2)	0.048 (2)	0.0351 (18)	−0.0036 (17)	−0.0031 (19)	0.0034 (19)
C3	0.044 (2)	0.047 (2)	0.039 (2)	−0.0027 (18)	−0.0130 (18)	0.0045 (17)
C4	0.042 (2)	0.054 (2)	0.042 (2)	−0.0125 (19)	−0.0049 (17)	0.0052 (18)
C5	0.046 (2)	0.064 (3)	0.035 (2)	−0.012 (2)	−0.0022 (18)	0.0027 (19)
C6	0.042 (2)	0.054 (2)	0.0337 (19)	−0.0073 (19)	−0.0050 (17)	0.0015 (17)
C7	0.0355 (18)	0.050 (2)	0.044 (2)	0.0016 (16)	0.0002 (18)	0.0066 (19)
C8	0.057 (2)	0.053 (2)	0.040 (2)	−0.009 (2)	−0.009 (2)	−0.0020 (17)
C9	0.029 (3)	0.071 (4)	0.051 (4)	0.017 (3)	0.019 (3)	0.002 (3)
C10	0.067 (5)	0.094 (6)	0.060 (5)	0.009 (5)	0.023 (4)	0.029 (5)
C11	0.061 (5)	0.099 (7)	0.060 (5)	0.016 (5)	0.003 (4)	0.040 (5)
C12	0.085 (7)	0.084 (6)	0.059 (5)	0.027 (5)	0.026 (5)	0.028 (5)
C13	0.125 (5)	0.125 (5)	0.125 (5)	0.0007 (10)	−0.0007 (10)	0.0000 (10)
N1	0.036 (2)	0.069 (4)	0.040 (3)	0.011 (3)	0.010 (2)	0.004 (3)
N2	0.035 (3)	0.083 (5)	0.060 (4)	0.013 (3)	0.022 (3)	0.016 (4)
C14	0.057 (3)	0.055 (3)	0.057 (3)	0.008 (2)	0.017 (2)	0.003 (2)
C15	0.066 (3)	0.092 (4)	0.043 (2)	0.004 (2)	0.007 (2)	−0.001 (2)
C16	0.061 (3)	0.061 (3)	0.059 (3)	−0.006 (2)	−0.016 (2)	0.003 (2)
C17	0.070 (4)	0.103 (5)	0.100 (4)	−0.031 (3)	−0.032 (3)	−0.002 (3)
C18	0.131 (3)	0.131 (3)	0.131 (3)	0.0004 (10)	0.0001 (10)	−0.0026 (10)
C19	0.041 (3)	0.101 (4)	0.084 (4)	0.015 (3)	0.029 (3)	0.004 (3)
C20	0.050 (3)	0.108 (4)	0.066 (3)	−0.007 (3)	0.015 (3)	0.017 (3)
C21	0.061 (3)	0.067 (3)	0.055 (3)	0.001 (3)	0.011 (2)	0.021 (2)
N3	0.054 (2)	0.067 (3)	0.062 (3)	0.0094 (18)	0.0164 (19)	0.015 (2)
N4	0.061 (2)	0.060 (2)	0.0451 (19)	0.005 (2)	0.0057 (18)	0.0053 (16)
O1	0.0416 (15)	0.0460 (16)	0.0479 (16)	−0.0031 (13)	−0.0066 (13)	0.0051 (13)
O2	0.070 (2)	0.089 (3)	0.0385 (18)	−0.025 (2)	−0.0001 (15)	0.0103 (16)
O3	0.097 (3)	0.109 (3)	0.066 (2)	−0.037 (3)	−0.003 (2)	−0.034 (3)
O4	0.0601 (19)	0.0649 (19)	0.0510 (18)	−0.0178 (15)	−0.0119 (16)	−0.0059 (18)
O5	0.080 (2)	0.104 (3)	0.0330 (16)	−0.041 (2)	0.0058 (16)	−0.0058 (17)

### Geometric parameters (Å, °)

**Table:**

Zn1—O1	1.992 (3)	C10'—C11'	1.38 (3)
Zn1—O4^i^	1.950 (3)	C10'—N2'	1.41 (3)
Zn1—N1	2.021 (5)	C10'—H10'	0.9300
Zn1—N1'	2.047 (13)	C11'—N1'	1.32 (2)
Zn1—N4^ii^	2.055 (4)	C11'—H11'	0.9300
C1—C6	1.386 (6)	C12'—C13'	1.50 (5)
C1—C2	1.390 (6)	C12'—H12C	0.9700
C1—C7	1.504 (6)	C12'—H12D	0.9700
C2—C3	1.378 (6)	C13'—H13D	0.9600
C2—H2	0.9300	C13'—H13E	0.9600
C3—C4	1.393 (6)	C13'—H13F	0.9600
C3—C8	1.517 (6)	N2'—C19	1.439 (16)
C4—C5	1.377 (6)	C14—N4	1.258 (6)
C4—H4	0.9300	C14—N3	1.341 (6)
C5—O5	1.367 (5)	C14—C17	1.529 (8)
C5—C6	1.390 (6)	C15—C16	1.331 (8)
C6—H6	0.9300	C15—N3	1.393 (7)
C7—O2	1.206 (5)	C15—H15	0.9300
C7—O1	1.311 (5)	C16—N4	1.380 (6)
C8—O3	1.225 (6)	C16—H16	0.9300
C8—O4	1.269 (6)	C17—C18	1.486 (11)
C9—N1	1.302 (9)	C17—H17A	0.9700
C9—N2	1.355 (9)	C17—H17B	0.9700
C9—C12	1.520 (11)	C18—H18A	0.9600
C10—C11	1.314 (12)	C18—H18B	0.9600
C10—N2	1.362 (12)	C18—H18C	0.9600
C10—H10	0.9300	C19—C20	1.520 (9)
C11—N1	1.388 (10)	C19—H19A	0.9700
C11—H11	0.9300	C19—H19B	0.9700
C12—C13	1.423 (17)	C20—C21	1.518 (8)
C12—H12A	0.9700	C20—H20A	0.9700
C12—H12B	0.9700	C20—H20B	0.9700
C13—H13A	0.9600	C21—N3	1.434 (6)
C13—H13B	0.9600	C21—H21A	0.9700
C13—H13C	0.9600	C21—H21B	0.9700
N2—C19	1.477 (8)	N4—Zn1^iii^	2.055 (4)
C9'—N1'	1.33 (2)	O4—Zn1^iv^	1.950 (3)
C9'—N2'	1.35 (2)	O5—H5	0.8200
C9'—C12'	1.46 (2)		
			
O4^i^—Zn1—O1	126.04 (14)	C9'—C12'—C13'	111 (2)
O4^i^—Zn1—N1	94.5 (2)	C9'—C12'—H12C	109.3
O1—Zn1—N1	119.9 (2)	C13'—C12'—H12C	109.3
O4^i^—Zn1—N1'	116.9 (4)	C9'—C12'—H12D	109.3
O1—Zn1—N1'	98.3 (4)	C13'—C12'—H12D	109.3
O4^i^—Zn1—N4^ii^	111.58 (15)	H12C—C12'—H12D	108.0
O1—Zn1—N4^ii^	93.26 (15)	C12'—C13'—H13D	109.5
N1—Zn1—N4^ii^	112.4 (2)	C12'—C13'—H13E	109.5
N1'—Zn1—N4^ii^	107.2 (4)	H13D—C13'—H13E	109.5
C6—C1—C2	120.3 (4)	C12'—C13'—H13F	109.5
C6—C1—C7	121.0 (4)	H13D—C13'—H13F	109.5
C2—C1—C7	118.6 (4)	H13E—C13'—H13F	109.5
C3—C2—C1	119.3 (4)	C11'—N1'—C9'	110.2 (15)
C3—C2—H2	120.3	C11'—N1'—Zn1	120.5 (13)
C1—C2—H2	120.3	C9'—N1'—Zn1	129.2 (13)
C2—C3—C4	120.7 (4)	C9'—N2'—C10'	108.6 (16)
C2—C3—C8	118.5 (4)	C9'—N2'—C19	133.6 (17)
C4—C3—C8	120.7 (4)	C10'—N2'—C19	117.7 (14)
C5—C4—C3	119.6 (4)	N4—C14—N3	114.4 (5)
C5—C4—H4	120.2	N4—C14—C17	123.3 (5)
C3—C4—H4	120.2	N3—C14—C17	121.8 (5)
O5—C5—C4	124.2 (4)	C16—C15—N3	106.6 (5)
O5—C5—C6	115.6 (4)	C16—C15—H15	126.7
C4—C5—C6	120.2 (4)	N3—C15—H15	126.7
C1—C6—C5	119.7 (4)	C15—C16—N4	109.3 (5)
C1—C6—H6	120.1	C15—C16—H16	125.4
C5—C6—H6	120.1	N4—C16—H16	125.4
O2—C7—O1	123.2 (4)	C18—C17—C14	112.4 (6)
O2—C7—C1	121.1 (4)	C18—C17—H17A	109.1
O1—C7—C1	115.7 (4)	C14—C17—H17A	109.1
O3—C8—O4	123.6 (4)	C18—C17—H17B	109.1
O3—C8—C3	119.7 (4)	C14—C17—H17B	109.1
O4—C8—C3	116.8 (4)	H17A—C17—H17B	107.8
N1—C9—N2	111.3 (8)	C17—C18—H18A	109.5
N1—C9—C12	123.7 (7)	C17—C18—H18B	109.5
N2—C9—C12	124.9 (7)	H18A—C18—H18B	109.5
C11—C10—N2	108.2 (7)	C17—C18—H18C	109.5
C11—C10—H10	125.9	H18A—C18—H18C	109.5
N2—C10—H10	125.9	H18B—C18—H18C	109.5
C10—C11—N1	109.1 (8)	N2'—C19—C20	106.6 (7)
C10—C11—H11	125.4	N2—C19—C20	117.4 (5)
N1—C11—H11	125.4	N2'—C19—H19A	85.2
C13—C12—C9	113.6 (10)	N2—C19—H19A	108.0
C13—C12—H12A	108.9	C20—C19—H19A	108.0
C9—C12—H12A	108.9	N2'—C19—H19B	137.0
C13—C12—H12B	108.9	N2—C19—H19B	108.0
C9—C12—H12B	108.9	C20—C19—H19B	108.0
H12A—C12—H12B	107.7	H19A—C19—H19B	107.2
C12—C13—H13A	109.5	C21—C20—C19	114.0 (5)
C12—C13—H13B	109.5	C21—C20—H20A	108.7
H13A—C13—H13B	109.5	C19—C20—H20A	108.7
C12—C13—H13C	109.5	C21—C20—H20B	108.7
H13A—C13—H13C	109.5	C19—C20—H20B	108.7
H13B—C13—H13C	109.5	H20A—C20—H20B	107.6
C9—N1—C11	105.5 (6)	N3—C21—C20	114.6 (4)
C9—N1—Zn1	134.0 (6)	N3—C21—H21A	108.6
C11—N1—Zn1	120.5 (5)	C20—C21—H21A	108.6
C9—N2—C10	105.7 (6)	N3—C21—H21B	108.6
C9—N2—C19	127.1 (8)	C20—C21—H21B	108.6
C10—N2—C19	126.3 (6)	H21A—C21—H21B	107.6
N1'—C9'—N2'	107.5 (18)	C14—N3—C15	104.2 (4)
N1'—C9'—C12'	129.8 (18)	C14—N3—C21	130.9 (5)
N2'—C9'—C12'	122.7 (17)	C15—N3—C21	124.9 (5)
C11'—C10'—N2'	103.9 (16)	C14—N4—C16	105.5 (4)
C11'—C10'—H10'	128.0	C14—N4—Zn1^iii^	134.6 (4)
N2'—C10'—H10'	128.0	C16—N4—Zn1^iii^	119.7 (3)
N1'—C11'—C10'	109.4 (18)	C7—O1—Zn1	109.3 (3)
N1'—C11'—H11'	125.3	C8—O4—Zn1^iv^	111.2 (3)
C10'—C11'—H11'	125.3	C5—O5—H5	109.5
			
C6—C1—C2—C3	3.6 (6)	C12'—C9'—N1'—Zn1	−5(3)
C7—C1—C2—C3	−172.8 (4)	O4^i^—Zn1—N1'—C11'	173.2 (12)
C1—C2—C3—C4	−3.2 (6)	O1—Zn1—N1'—C11'	−49.0 (13)
C1—C2—C3—C8	174.6 (4)	N1—Zn1—N1'—C11'	153.7 (19)
C2—C3—C4—C5	0.5 (7)	N4^ii^—Zn1—N1'—C11'	47.1 (14)
C8—C3—C4—C5	−177.3 (4)	O4^i^—Zn1—N1'—C9'	−2.2 (15)
C3—C4—C5—O5	−179.0 (5)	O1—Zn1—N1'—C9'	135.6 (13)
C3—C4—C5—C6	2.0 (7)	N1—Zn1—N1'—C9'	−21.7 (11)
C2—C1—C6—C5	−1.3 (6)	N4^ii^—Zn1—N1'—C9'	−128.3 (13)
C7—C1—C6—C5	175.1 (4)	N1'—C9'—N2'—C10'	2.9 (19)
O5—C5—C6—C1	179.3 (4)	C12'—C9'—N2'—C10'	−176.5 (16)
C4—C5—C6—C1	−1.6 (7)	N1'—C9'—N2'—C19	−176.2 (15)
C6—C1—C7—O2	−161.1 (4)	C12'—C9'—N2'—C19	4(3)
C2—C1—C7—O2	15.2 (6)	C11'—C10'—N2'—C9'	−4.8 (19)
C6—C1—C7—O1	17.3 (6)	C11'—C10'—N2'—C19	174.5 (13)
C2—C1—C7—O1	−166.3 (4)	N3—C15—C16—N4	2.4 (6)
C2—C3—C8—O3	21.4 (7)	N4—C14—C17—C18	65.8 (8)
C4—C3—C8—O3	−160.7 (5)	N3—C14—C17—C18	−105.1 (7)
C2—C3—C8—O4	−158.8 (4)	C9'—N2'—C19—N2	31.4 (15)
C4—C3—C8—O4	19.0 (6)	C10'—N2'—C19—N2	−148 (2)
N2—C10—C11—N1	1.1 (12)	C9'—N2'—C19—C20	−84.1 (19)
N1—C9—C12—C13	−85.8 (13)	C10'—N2'—C19—C20	96.9 (14)
N2—C9—C12—C13	90.9 (13)	C9—N2—C19—N2'	1.7 (13)
N2—C9—N1—C11	−3.2 (10)	C10—N2—C19—N2'	−166.2 (18)
C12—C9—N1—C11	173.9 (9)	C9—N2—C19—C20	78.4 (10)
N2—C9—N1—Zn1	179.5 (6)	C10—N2—C19—C20	−89.5 (10)
C12—C9—N1—Zn1	−3.4 (12)	N2'—C19—C20—C21	−164.5 (8)
C10—C11—N1—C9	1.3 (11)	N2—C19—C20—C21	163.6 (5)
C10—C11—N1—Zn1	179.0 (7)	C19—C20—C21—N3	−55.8 (7)
O4^i^—Zn1—N1—C9	−165.9 (7)	N4—C14—N3—C15	2.7 (6)
O1—Zn1—N1—C9	−29.4 (8)	C17—C14—N3—C15	174.3 (5)
N1'—Zn1—N1—C9	−3.3 (10)	N4—C14—N3—C21	−179.8 (5)
N4^ii^—Zn1—N1—C9	78.6 (7)	C17—C14—N3—C21	−8.2 (8)
O4^i^—Zn1—N1—C11	17.1 (7)	C16—C15—N3—C14	−3.0 (6)
O1—Zn1—N1—C11	153.7 (6)	C16—C15—N3—C21	179.3 (5)
N1'—Zn1—N1—C11	179.7 (13)	C20—C21—N3—C14	−78.2 (7)
N4^ii^—Zn1—N1—C11	−98.4 (7)	C20—C21—N3—C15	98.8 (6)
N1—C9—N2—C10	3.9 (10)	N3—C14—N4—C16	−1.2 (6)
C12—C9—N2—C10	−173.2 (9)	C17—C14—N4—C16	−172.7 (5)
N1—C9—N2—C19	−166.0 (7)	N3—C14—N4—Zn1^iii^	−176.4 (3)
C12—C9—N2—C19	17.0 (14)	C17—C14—N4—Zn1^iii^	12.0 (8)
C11—C10—N2—C9	−3.0 (11)	C15—C16—N4—C14	−0.8 (6)
C11—C10—N2—C19	167.0 (9)	C15—C16—N4—Zn1^iii^	175.3 (4)
N2'—C10'—C11'—N1'	4.9 (19)	O2—C7—O1—Zn1	17.5 (5)
N1'—C9'—C12'—C13'	108 (3)	C1—C7—O1—Zn1	−161.0 (3)
N2'—C9'—C12'—C13'	−73 (3)	O4^i^—Zn1—O1—C7	33.0 (3)
C10'—C11'—N1'—C9'	−3(2)	N1—Zn1—O1—C7	−89.0 (3)
C10'—C11'—N1'—Zn1	−179.6 (11)	N1'—Zn1—O1—C7	−99.2 (4)
N2'—C9'—N1'—C11'	0.3 (19)	N4^ii^—Zn1—O1—C7	152.8 (3)
C12'—C9'—N1'—C11'	179.6 (18)	O3—C8—O4—Zn1^iv^	−10.0 (6)
N2'—C9'—N1'—Zn1	176.1 (11)	C3—C8—O4—Zn1^iv^	170.2 (3)

Symmetry codes: (i) −*x*, *y*+1/2, −*z*+3/2; (ii) −*x*+3/2, −*y*+1, *z*+1/2; (iii) −*x*+3/2, −*y*+1, *z*−1/2; (iv) −*x*, *y*−1/2, −*z*+3/2.

### Hydrogen-bond geometry (Å, °)

**Table:**

*D*—H···*A*	*D*—H	H···*A*	*D*···*A*	*D*—H···*A*
O5—H5···O1^v^	0.82	1.97	2.741 (5)	156

Symmetry codes: (v) *x*−1/2, −*y*+1/2, −*z*+2.
